# Increased short-term mortality among normothermic patients presenting to a medical emergency department with infection - a cohort study

**DOI:** 10.1186/1757-7241-23-S1-A26

**Published:** 2015-07-16

**Authors:** Daniel P Henriksen, Helene CK Jensen, Christian B Laursen, Annmarie T Lassen

**Affiliations:** 1 Medical Emergency Department, OUH Odense University Hospital, Odense, Denmark; 2 Department of Respiratory Medicine, OUH Odense University Hospital, Odense, Denmark

## Background

Infections are frequent causes of medical admissions to the emergency department (ED). However, not all infected patients present with fever (>38°C). The aim of the study was to assess differences in short-term mortality among patients hospitalized with community-acquired infection presenting with and without fever.

## Methods

All adult patients (≥15 years) with a first-time admission at a medical ED between September 2010-August 2011 with community-acquired infection were included. Cases were identified by manual chart review using predefined criteria of infection. Data on vital signs, laboratory values and antibiotic treatment were obtained electronically. We excluded unidentified patients and patients residing outside the hospitals catchment area. To assess if the absence of fever (normothermia: 36.0-38.0°C) is an independent prognostic factor, we computed multiple Cox regression analyses, adjusted for different potential confounders.

## Results

1,901 patients with infection, treated with antibiotics within 24 hours after arrival, were included. Median age was 74 years (5-95% range: 29-92 years); 896 (47.1%) were males, and 811 (42.7%) presented with a Charlson Comorbidity Index >2; 49.7% were normothermic at arrival and 50.3% presented with fever. Thirty-day mortality was 9.3% (95% CI: 7.5-11.3%) among patients with fever and 18.1% (95% CI: 15.7-20.7%) in normothermic patients. The unadjusted hazard ratio (HR) of death within 30 days after admission in normothermic infectious patients was 2.2 (95% CI: 1.7-2.8) compared to infectious patients with fever, adjusted HR 2.0 (95% CI: 1.6-2.6).

## Conclusion

Normothermic patients admitted with an infection were twice as likely to die within 30 days after admission compared to infectious patients admitted with fever.

